# Single incision VATS decortication for 3rd stage empyema

**DOI:** 10.1002/ccr3.1743

**Published:** 2018-09-25

**Authors:** Yasser Aljehani, Sukainah Alabkary

**Affiliations:** ^1^ Thoracic Surgery Division Department of Surgery College of Medicine King Fahad Hospital of the University Imam Abdulrahman Bin Faisal University Al‐Khobar, Dammam Saudi Arabia

**Keywords:** decortication, empyema, uniportal, VATS

## Abstract

The use of U‐VATS should be preferred to conventional three‐port VATS if skills are mastered as it causes less and single intercostal trauma. Moreover, it minimizes infection spread to the chest wall. We advocate the use of U‐VATS as a routine approach in patient with empyema stage III for positive outcome.

## 1 MATERIAL & METHOD

This is a single case report.

## 2 THE CASE

**Figure 1:**
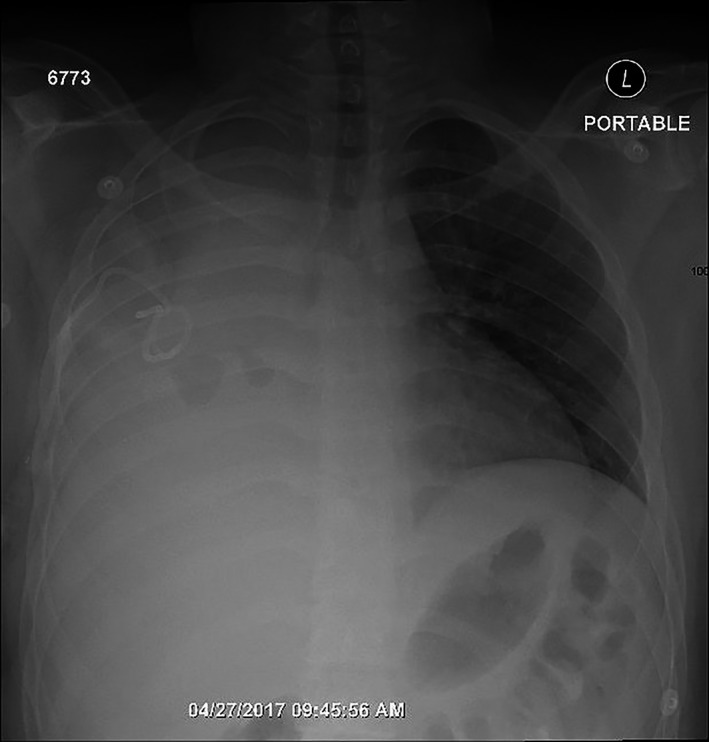
Chest x‐ray showing right hemi‐thorax opacification

**Figure 2:**
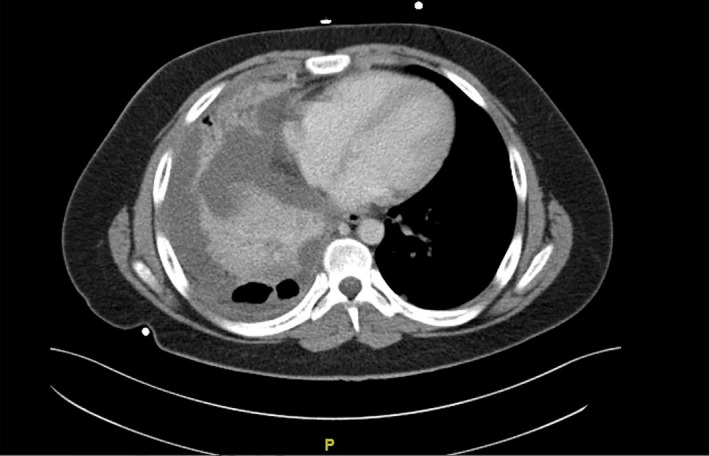
Mediastinal view of CT chest showing right‐sided pleural effusion

A 17‐year‐old male known case of bronchial asthma and recently diagnosed as a case of diabetes mellitus on insulin therapy. The patient was referred with a history of right shoulder pain for 4 days duration. The pain was pleuritic in nature and got worse in the last 2 days. The pain was associated with fever, dyspnea, and productive cough. He had parapneumonic effusion for which pigtail catheter was inserted but minimal drainage achieved. On arrival, the patient was looking ill, tachypneic, febrile, with right shoulder, and chest pain. Blood pressure: 127/72 mmHg, Pulse: 124/min, S_o2_: 92% in room air, Temperature: 38.1^°^. Chest auscultation revealed bronchial breath sound, and decrease air entry on the right side. Chest x‐ray showed complete right opacification of hemithorax (Figure 1). Computed tomography (CT) chest was performed and showed significant amount of right‐sided pleural effusion with pockets of air (possibly due to pigtail insertion), consolidation of right upper and lower lobes, and pleural thickening (Figure 2). The patient underwent right flexible bronchoscopy and right U‐VATS decortication. Under general anesthesia, double lumen endotracheal tube was inserted. Left decubitus position. The single port (2 cm) was introduced in the 7th intercostal space in posterior axillary line to access the subpulmonic area as well. The port position is not fixed point rather selected based on studying the CT scan and introducing the port through a safe area. We did not use wound protector as there was no fluid material to contaminate the wound. The protector would obstruct the introduction of instruments in this single incision. There were extensive adhesions between parietal and visceral pleura.

**Figure 3:**
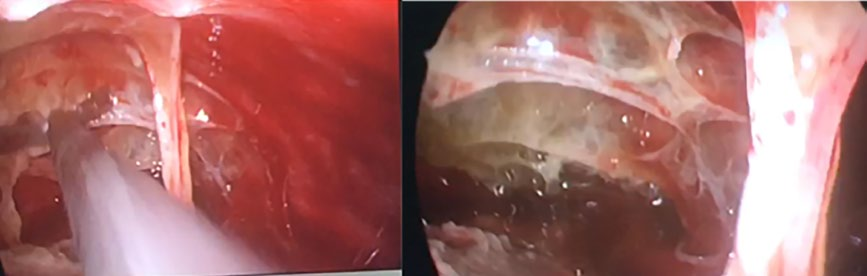
*Right sided U‐VATS and for stage III empyema using 2 cm single incision*

**Figure 4:**
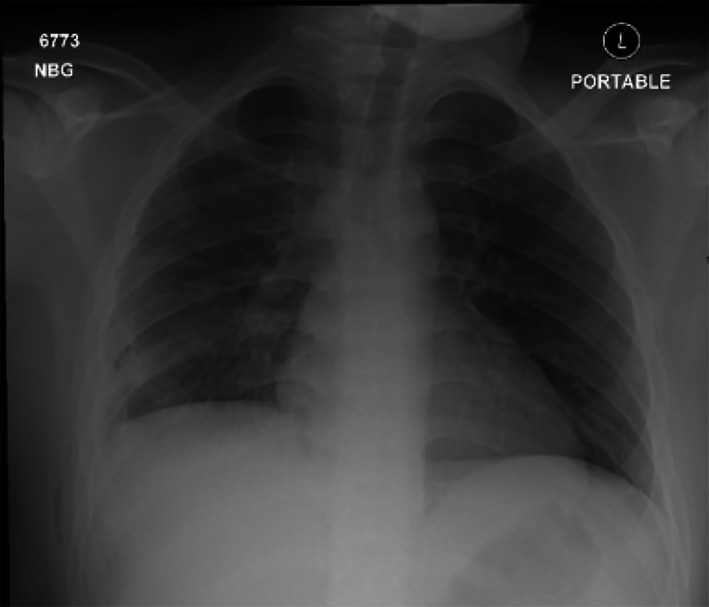
*Chest x‐ray showing fully expanded lungs*

A 5‐mm 30° camera was introduced and debris with multi‐loculations were encountered, thick peel over the visceral pleura was seen covering mainly the lower lobe. Loculations were lysed and debris removed using endoscopic soft bowel clamps and ovum forceps. Thickened peel over the visceral pleura was peeled off and lung was cleared completely from costal, mediastinal and diaphragmatic surfaces (Figure 3). The sub‐pulmonic space was approached through following the lung border at the mediastinal surfaces were the adhesions usually less. Adhesions were lysed completely from all three lobes. Complete freeing of all lobes and full lung expansion were achieved. Hemostasis insured and test for air leak was performed. Two thoracostomy tubes, size 28 F, were inserted, apically, and in the subpulmonic space through the single incision. The patient had an uneventful postoperative course and his thoracostomy tubes were removed in postoperative day 2 and 3 respectively. He was discharged home and seen in the clinic at 1 week, 1 month, and 3 months with complete resolution of his condition. His chest x‐ray upon discharge showed complete resolution (Figure 4).

## 3 DISCUSSION

Empyema thoracis is one of thoracic surgical entities that present with clinical challenges due to significant morbidity and even mortality. Its prevalence is increasing globally.^1^, ^2^ It most commonly occurs in the setting of bacterial pneumonia. Fifty to 70% of patients with pneumonia will develop parapneumonic effusion (Stage I), and 20% of them will develop pleural empyema in a fibrinopurulent or organized stage (stage II or III).^1^, ^3^

The initial management of pleural empyema is antibiotic treatment and pleural drainage. It is insufficient in stage II or stage III empyema, due to fibrin deposit produces thick pleural peel and loculated pleural fluid. This peel causes lung restriction (also termed trapped lung). Furthermore, surgical intervention is needed in such cases.^1^, ^3^ Open or VATS pleural decortication is an effective initial approach for advanced empyema. They are both equally effective as they allow to achieve the essential steps for full lung expansion, decortication of pleural peels, disruption of loculation, and fluid evacuation.^3^, ^4^ Early surgical intervention gives a better chance of success in VATS decortication than open decortication especially in stage II. We have to differentiate between deloculation and decortication, as many consider deloculation as decortication. Deloculation is simple and straightforward and VATS is the option for it. Decortication of the thick peel is difficult and needs skilled thoracic surgeon especially through VATS. In agreement with many reports, VATS decortication is feasible and safe alternative, as it provides a better visualization of the entire pleural cavity. It is associated with lower morbidity rate, lower cost, shorter hospital stay, and good postoperative functional, and cosmetic outcomes compared to standard thoracotomy approach.^3^, ^5^ Obliterated pleural space is a major obstacle in preforming VATS and the conversion to conventional open decortication is considered in many cases.^3^

The recent modification to VATS is uniportal VATS (U‐VATS). It is preferred to conventional three ports VATS when it comes to morbidity and patient comfort. This leads to single intercostal trauma, better cosmetics, and minimal infection spread to the chest wall in patient with empyema.^5^, ^6^ In U‐VATS, multiple endoscopic instruments and the camera are inserted through a single incision without spreading the ribs. The incision ranges between 2 and 8 cm single skin incisions. In our case, we used 2 cm uniportal VATS. There are two most common limitations for this technique: the crowding of surgical instruments and the optical lens getting dirty easily. For this reason, it is necessary that the new generation of thoracic surgeons get trained to use uniportal technique.^2^ The author performed several cases and the results are promising.

## 4 CONCLUSION

In conclusion, early referral to thoracic surgery gives a better chance to VATS decortication for empyema in stage III. The use of U‐VATS should be preferred to conventional three‐port VATS if skills are mastered as it causes less and single intercostal trauma. Moreover, it minimizes infection spread to the chest wall. We advocate the use of U‐VATS as a routine approach in patient with empyema stage III for positive outcomes.

## CONFLICT OF INTEREST

None declared.

## AUTHORSHIP

YMA: designed the study, revised the manuscript critically for important intellectual content, and approved the version of the manuscript to be published. SHA: drafted the manuscript, analyzed the data, and approved the version of the manuscript to be published. We have not received substantial contributions from nonauthors.
